# Combined Striatal Dopaminergic and Cardiac Sympathetic Imaging in Parkinson’s Disease

**DOI:** 10.3390/brainsci16050484

**Published:** 2026-04-30

**Authors:** Louis Versweyveld, Greet Vanderlinden, Wim Vandenberghe, Aline Delva, Koen Van Laere

**Affiliations:** 1 Nuclear Medicine and Molecular Imaging, Department of Imaging and Pathology, Leuven Brain Institute, KU Leuven, 3000 Leuven, Belgium; louis.versweyveld@uzleuven.be (L.V.); greet.vanderlinden@kuleuven.be (G.V.); 2 Department of Neurology, University Hospitals Leuven, 3000 Leuven, Belgium; wim.vandenberghe@uzleuven.be (W.V.); aline.delva@uzleuven.be (A.D.); 3 Department of Neurosciences, Leuven Brain Institute, KU Leuven, 3000 Leuven, Belgium; 4 Division of Nuclear Medicine, University Hospitals Leuven, 3000 Leuven, Belgium

**Keywords:** Parkinson’s disease, molecular imaging, scintigraphy, SPECT

## Abstract

**Background/Objectives:** Imaging of both cardiac sympathetic denervation and nigrostriatal dopaminergic degeneration can support the diagnosis of Parkinson’s disease (PD). However, their temporal relationship and combined diagnostic value remain unclear. This review addresses (1) whether nigrostriatal degeneration and cardiac sympathetic denervation are correlated in PD and (2) the comparative and combined diagnostic accuracy of striatal dopaminergic and cardiac sympathetic imaging in PD. **Methods:** We searched PubMed (October 2025) for studies assessing both a striatal dopaminergic and a cardiac sympathetic imaging biomarker in the same PD cohort, supplemented by citation chaining. Diagnostic accuracy studies were evaluated using QUADAS-2. **Results:** Nineteen studies met the inclusion criteria. Ten studies examined within-subject associations; six reported significant correlations ranging from weak to strong (ρ ~0.2–0.8). Two studies observed a significant correlation for the akinetic-rigid subtype but not for the tremor-dominant subtype of PD. Ten studies compared diagnostic accuracies, five of which used pre-defined thresholds and consistently found higher sensitivity for [^123^I]FP-CIT SPECT (78–100%) compared to [^123^I]MIBG scintigraphy (65–82%), but higher specificity for [^123^I]MIBG (range 75–100%) than for [^123^I]FP-CIT (range 11–73%). Adding [^123^I]MIBG scintigraphy to [^123^I]FP-CIT SPECT generally increased specificity but had inconsistent effects on overall accuracy. QUADAS-2 revealed substantial risks of patient selection bias, data-driven thresholds, and limited blinding. **Conclusions:** Reported correlations between nigrostriatal dopaminergic degeneration and cardiac sympathetic denervation in PD are inconsistent, likely reflecting both methodological heterogeneity and real variation between phenotypes. There may be a stronger correlation in the akinetic-rigid phenotype. Dopaminergic imaging is more sensitive in early PD, while cardiac sympathetic imaging is more specific for differentiating PD from atypical Parkinsonian syndromes. However, study designs greatly restrict the generalizability of reported diagnostic accuracies.

## 1. Introduction

Parkinson’s disease (PD) is a neurodegenerative condition defined by the loss of dopaminergic neurons in the substantia nigra pars compacta and the accumulation of α-synuclein in Lewy bodies and Lewy neurites [1]. The clinical diagnosis of PD is based on the cardinal motor symptoms of bradykinesia, rigidity and tremor, but the accuracy of clinical diagnosis is lower in the first years [2].

Some early pathophysiological aspects present an opportunity for the use of molecular imaging biomarker techniques in clinical practice. Parkinsonian motor symptoms only appear approximately 8 years after striatal synaptic dopamine transporter (DAT) availability begins to decline [3] and a threshold of approximately 70% loss of dopaminergic synapses in the striatum has been reached [4]. Cardiac sympathetic denervation can even occur before nigrostriatal dopaminergic degeneration in isolated rapid eye movement sleep behavior disorder (iRBD), a parasomnia that frequently precedes PD or other α-synucleinopathies [5]. The onset of cardiac sympathetic denervation relative to nigrostriatal dopaminergic degeneration has been observed to be very variable in PD [6]. In theory, this could imply a weak within-subject correlation of both biomarkers and a complementary sensitivity for different subtypes of PD.

**Figure 1:**
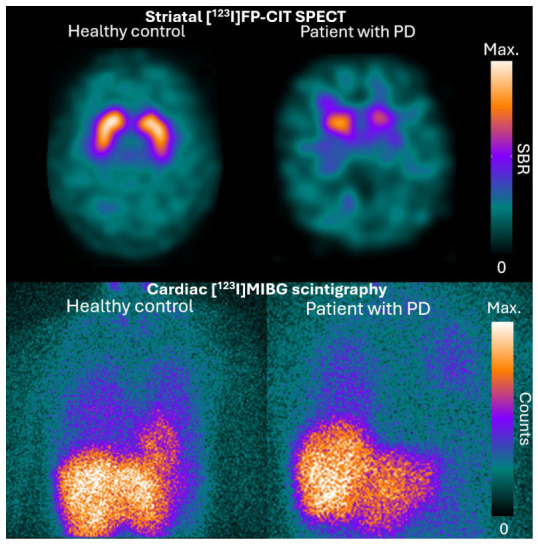
Typical examples of striatal [^123^I]FP-CIT SPECT and cardiac [^123^I]MIBG scintigraphy. SBR = Specific Binding Ratio.

In clinical practice, SPECT with ^123^I-ioflupane ([^123^I]FP-CIT), a DAT radioligand, is commonly used for the differentiation of PD from non-degenerative forms of Parkinsonism, as these conditions typically show preserved DAT binding, in contrast to the reduced DAT binding observed in PD. The current Movement Disorders Society (MDS) diagnostic criteria for PD define normal DAT imaging of the presynaptic dopaminergic system as an absolute exclusion criterion [7]. However, uptake is often also reduced in multiple system atrophy (MSA) and progressive supranuclear palsy (PSP), so [^123^I]FP-CIT SPECT cannot be used to differentiate PD from atypical Parkinsonian syndromes (APS) [8]. ^123^I-metaiodobenzylguanidine ([^123^I]MIBG) scintigraphy is used to evaluate cardiac innervation by targeting the norepinephrine transporter (NET) of the post-ganglionic sympathetic nerves. It is defined as a supportive biomarker criterion for the diagnosis of PD by the MDS diagnostic criteria because of high specificity for the differentiation between PD and APS [7,9]. Illustrative examples of a normal and abnormal striatal [^123^I]FP-CIT SPECT and cardiac [^123^I]MIBG scintigraphy are provided in Figure 1.

The clinical setting where cardiac [^123^I]MIBG scintigraphy adds value is still unclear, since most studies had small sample sizes and/or variable design, methodology, and population. Furthermore, only a few included pathological validation [9]. In this review, we summarize studies published in the last 20 years reporting a combined use of striatal dopaminergic and cardiac sympathetic imaging in patients with PD. We aim to summarize the available evidence on the within-subject correlation of nigrostriatal degeneration and cardiac sympathetic denervation, and assess how strongly the evidence supports a complementary diagnostic role of striatal dopaminergic and cardiac sympathetic imaging in PD.

## 2. Materials and Methods

**Figure 2:**
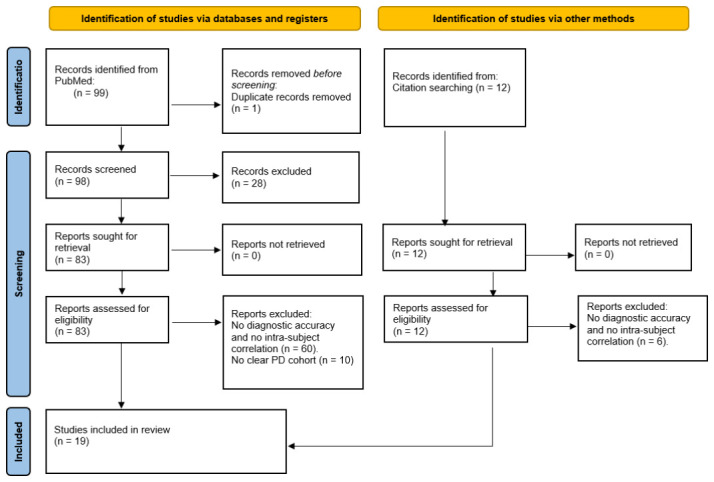
PRISMA flow diagram of study selection.

We searched PubMed in October 2025 using tracer-based search terms covering PET and SPECT markers of nigrostriatal dopaminergic integrity, including tracers of presynaptic terminal density (DAT and VMAT2 tracers) and dopamine synthesis capacity ([^18^F]-FDOPA), as well as markers of post-ganglionic cardiac sympathetic innervation ([^123^I]-MIBG and [^11^C]-HED). The full search strategy, including all keywords and Boolean operators, is provided in the Supplementary Materials. Studies were eligible if they reported imaging results for at least one imaging biomarker of striatal dopaminergic degeneration and at least one imaging biomarker of cardiac sympathetic denervation acquired in the same cohort of patients with PD. From the eligible studies, two types were selected for further analysis: (1) studies that reported on the within-subject correlation between striatal dopaminergic and cardiac sympathetic imaging, and (2) studies that reported on the diagnostic accuracy of striatal dopaminergic and cardiac sympathetic imaging to differentiate patients with PD from a comparison group. Case reports, conference abstracts, non-original articles, and studies published before 2005 were excluded. After screening PubMed search results by title and abstract, potentially relevant articles were assessed in full text. Citation chaining was performed using an online citation-mapping tool (ResearchRabbit) to identify studies missed by the initial database search. These studies underwent the same eligibility assessment. The study selection process is summarized in a PRISMA 2020 flow diagram in Figure 2 [10].

**Figure 3:**
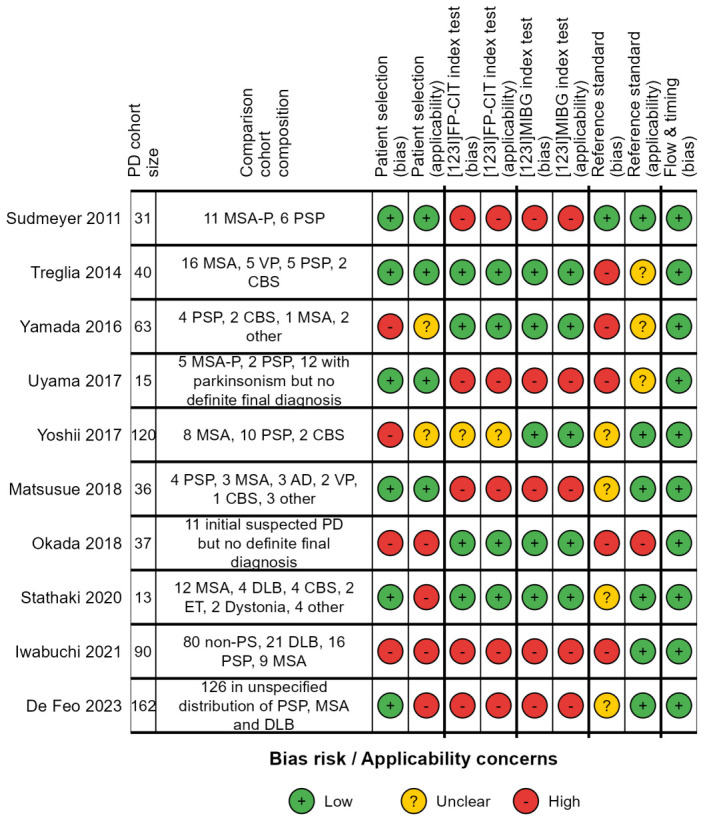
Qualitative analysis of included studies that report diagnostic accuracy by Quality Assessment Tool for Diagnostic Accuracy Studies-2 (QUADAS-2) [12,13,14,15,16,17,18,19,20,21]. Applicability concerns are specific to this review and do not reflect the quality of studies. Abbreviations: MSA-P multiple systems atrophy–Parkinsonian variant, PSP progressive supranuclear palsy, VP vascular Parkinsonism, CBS corticobasal syndrome, AD Alzheimer’s dementia, non-PS non-Parkinsonian syndrome, DLB dementia with Lewy bodies.

To compare the diagnostic accuracy of striatal dopaminergic and cardiac sympathetic imaging in PD, a selection was made of studies that reported the sensitivity and specificity of ^123^I-FP-CIT SPECT and [^123^I]MIBG scintigraphy or SPECT in a cohort of patients with PD and a comparison group. Using the QUADAS-2 criteria [11], we constructed a traffic light chart to visualize the risk of bias and concerns regarding applicability to this review (Figure 3).

## 3. Results

### 3.1. Study Selection and Study Characteristics

We identified 19 eligible studies. Cardiac sympathetic imaging was done with [^123^I]MIBG scintigraphy in 18 studies and with [^11^C]HED PET in one study. Striatal dopaminergic imaging was performed with [^123^I]FP-CIT SPECT in 14 studies, with additional studies using [^18^F]FP-CIT PET (*n* = 2), [^11^C]DTBZ PET (*n* = 1), and [^11^C]CFT PET (*n* = 1). Of the included studies, ten assessed within-subject correlations, ten reported diagnostic accuracy, and eight evaluated combined imaging approaches.

### 3.2. Within-Subject Correlations Between Striatal Dopaminergic and Cardiac Sympathetic Measures in PD

Ten of the 19 included studies explicitly reported within-subject correlations between nigrostriatal dopaminergic degeneration and cardiac sympathetic denervation in PD. The subject characteristics, imaging modalities, and uptake parameters are summarized in Supplementary Table S1. Six studies reported significant correlations between cardiac [^123^I]MIBG uptake and striatal DAT imaging (two ^18^F-FP-CIT PET and four ^123^I-FP-CIT SPECT) in a PD cohort [12,22,23,24,25,26].

Two other studies using [^123^I]MIBG scintigraphy and ^123^I-FP-CIT SPECT found a correlation only in the akinetic-rigid subgroup of 14 out of 31 [27] and 29 out of 40 [28] patients with PD, but not in the tremor-dominant subgroup. The other studies did not report separate results per phenotype.

Two studies reported no correlation, with one between cardiac [^11^C]HED retention and striatal [^11^C]DTBZ binding [29], and the other between cardiac [^123^I]MIBG uptake and striatal [^11^C]CFT uptake [30]. Importantly, these two studies had small sample sizes of 9 and 16 patients with PD, respectively, whereas the eight [^123^I]MIBG-DAT studies had larger sample sizes (average *n* = 59, range 15–120).

Spearman coefficients in the eight [^123^I]MIBG-DAT studies varied widely from weak (ρ ≈ 0.23–0.40) [12,22,23,26] to strong (ρ ≈ 0.63–0.79) [24,27], or were not reported. The studies with the largest sample sizes showed weak but statistically significant Spearman correlations (*n* = 120, ρ = 0.23, *p* = 0.02 [12]; *n* = 96, ρ = 0.36, *p* < 0.01 [22]).

Of note, two studies found a linear correlation independently of age, disease duration, and clinical severity [22,23]. Imaging techniques varied across studies, mainly with regard to DAT imaging modality (^18^F-FP-CIT PET vs. ^123^I-FP-CIT SPECT) and region-of-interest definition (caudate, putamen, whole striatum, ipsilateral, contralateral, or both).

### 3.3. Diagnostic Accuracy of Striatal DAT Versus Cardiac Sympathetic Imaging

Ten of the included studies explicitly reported the diagnostic accuracy of both cardiac sympathetic imaging with [^123^I]MIBG scintigraphy and nigrostriatal dopaminergic denervation with [^123^I]FP-CIT SPECT. Comparison groups were completely or mostly composed of APS in seven of ten studies, and included Parkinsonism patients with unspecified final diagnosis in three studies.

Across eight studies that reported sensitivity and specificity, sensitivity for detecting PD ranged from 65 to 93% for [^123^I]MIBG and 69 to 100% for [^123^I]FP-CIT. Specificity for differentiating PD from the comparison groups ranged from 47% to 100% for [^123^I]MIBG scintigraphy and from 11% to 75% for [^123^I]FP-CIT SPECT.

In a subset of five studies using pre-defined thresholds [12,13,14,15,16], sensitivity was consistently higher for [^123^I]FP-CIT (78–100%) compared to [^123^I]MIBG (65–82%), while specificity was always higher for [^123^I]MIBG (range 75–100%) than for [^123^I]FP-CIT (range 11–73%). Of note, one study reported a 100% specificity for [^123^I]MIBG scintigraphy but included a comparison group of eleven patients with mild Parkinsonism and unclear diagnosis [15]. Another study reported a 100% sensitivity in 17 patients with PD for [^123^I]FP-CIT SPECT but did not report the threshold applied for semi-quantitative assessment [16].

One additional study reported diagnostic accuracy using a ROC analysis only. This study evaluated a large cohort of 288 patients with suspected de novo PD or non-PD Parkinsonian syndromes who underwent both [^123^I]MIBG scintigraphy and [^123^I]FP-CIT SPECT. After a three-year clinical follow-up, 162 patients were diagnosed with PD, while 126 were diagnosed as non-PD, including PSP, dementia with Lewy bodies (DLB), and MSA, but the exact distribution of PSP, DLB, and MSA was not specified [17]. The study reported a higher diagnostic accuracy to differentiate de novo PD from atypical Parkinsonism with [^123^I]MIBG scintigraphy (AUC = 0.94) than with [^123^I]FP-CIT SPECT (AUC = 0.56). The low AUC for [^123^I]FP-CIT SPECT likely reflects study-specific factors and should be interpreted cautiously.

### 3.4. Diagnostic Accuracy of Combined Striatal DAT and Cardiac Sympathetic Imaging

Eight of the included studies reported sensitivity and specificity for a combined assessment with both [^123^I]MIBG and [^123^I]FP-CIT and are summarized in Table 1. Combined approaches included rule-based combined positivity and multivariable and model-based combinations. The used uptake parameters and thresholds are summarized in Supplementary Table S2.

#### 3.4.1. Combined Positivity

Five studies reported the sensitivity and specificity of a positive result of both [^123^I]MIBG scintigraphy and [^123^I]FP-CIT SPECT [12,13,14,16,20] for the discrimination of PD from comparison groups consisting mostly of APS. The average sensitivity and specificity for differentiating PD from the comparison groups across studies were 93% and 28% for [^123^I]FP-CIT, 82% and 76% for [^123^I]MIBG, and 78% and 86% for a combined assessment. This suggests that requiring combined positivity for both modalities results in very high specificity, but at the cost of lower sensitivity compared to [^123^I]FP-CIT alone.

#### 3.4.2. Multivariable and Model-Based Combinations

Three studies combined semi-quantitative indices of striatal [^123^I]FP-CIT SPECT and cardiac [^123^I]MIBG scintigraphy for the diagnosis of PD. Okada et al. compared the formula [^123^I]FP-CIT specific binding ratio (SBR) × [^123^I]MIBG heart-to-mediastinum ratio (H/M ratio) to each semi-quantitative index alone, and found a sensitivity of 81% and specificity of 91% for SBR × H/M ratio compared to a sensitivity of 78% and specificity of 73% for [^123^I]FP-CIT SBR alone [15]. One study combined several quantitative indices—including [^123^I]MIBG H/M ratios, washout-rate, and [^123^I]FP-CIT metrics—into a multiparametric scoring system (MSS) that assigned a score of 0 or 1 to all five parameters. In differentiating PD from APS, the MSS had the same sensitivity and specificity as the delayed [^123^I]MIBG H/M ratio alone. For PD versus non-PD, in a non-APS cohort composed of Alzheimer’s disease, vascular Parkinsonism, and other disorders, the MSS displayed a sensitivity of 83% and specificity of 88%, with superior diagnostic accuracy to individual parameters [18]. Another study developed a diagnostic decision tree using [^123^I]FP-CIT SPECT and [^123^I]MIBG scintigraphy quantitative indices, achieving a sensitivity of 91% and specificity of 79% for differentiating PD from non-PD disorders, but diagnostic accuracy for individual modalities or indices was not reported [19].

### 3.5. Risk of Bias and Applicability of Diagnostic Accuracy Studies (QUADAS-2)

#### 3.5.1. Patient Selection and Spectrum

A qualitative analysis of the ten studies reporting on diagnostic accuracy is shown in Figure 3. Four studies retrospectively selected subjects for a case–control design, introducing a high risk of selection bias, while six studies retrospectively included consecutive patients with suspected PD or Parkinsonism who had received both [^123^I]FP-CIT and [^123^I]MIBG with clear a priori exclusion criteria, implicating a lower risk of bias. In the latter six studies, including only patients who received both [^123^I]FP-CIT SPECT and [^123^I]MIBG scintigraphy likely led to an overrepresentation of APS, since clinicians are more likely to request both scans in patients with atypical symptoms than in patients with typical PD. This selection strategy likely inflated specificity estimates for cardiac imaging.

#### 3.5.2. Index Test

Five studies derived study-specific thresholds (cutoff values) for semi-quantification of [^123^I]FP-CIT SPECT and [^123^I]MIBG scintigraphy post hoc, introducing a high risk of bias and limiting the external validity of the reported diagnostic accuracies. In contrast, five studies used pre-defined cutoff values, which have a lower risk of bias but may pose a risk of inaccuracy unless similar SPECT acquisition and reconstruction techniques/camera systems are used. Most studies performed in Japan used the same software for semi-quantification of [^123^I]FP-CIT SPECT (DaTView, Nihon Medi-Physics, Tokyo, Japan) and the same striatal uptake parameter, whereas other studies used other, various software packages and uptake parameters (Supplementary Table S2).

#### 3.5.3. Diagnostic Reference Standard

All ten studies used clinical diagnosis as the reference standard, but only one explicitly stated that the final clinical diagnosis was made by an investigator blinded to the [^123^I]MIBG scintigraphy and [^123^I]FP-CIT SPECT results. Five studies reported no blinding but included a longitudinal clinical follow-up with assessment of dopaminergic treatment response. The timing of the clinical diagnosis relative to the imaging was not specified in most studies.

## 4. Discussion

### 4.1. Principal Findings

Across 19 eligible studies, evidence for a within-subject correlation between striatal dopaminergic degeneration and cardiac sympathetic denervation was inconsistent and heterogeneous. In diagnostic studies, striatal DAT imaging tended to be more sensitive, whereas [^123^I]MIBG scintigraphy tended to be more specific for differentiating PD from comparison groups with mainly APS. Combined assessments resulted in very high specificity in most studies, but with varying effects on sensitivity compared to [^123^I]FP-CIT alone.

### 4.2. Correlation Between Nigrostriatal Dopaminergic Degeneration and Cardiac Sympathetic Denervation

Out of ten studies assessing within-subject correlation between nigrostriatal dopaminergic degeneration and cardiac sympathetic denervation, six found a correlation between cardiac [^123^I]MIBG uptake and striatal DAT imaging. Two other studies, also using [^123^I]MIBG and DAT imaging, only found a correlation in the akinetic-rigid subgroups [27,28], and two small studies found no correlation between cardiac [^11^C]HED retention and striatal [^11^C]DTBZ binding [29] nor between cardiac [^123^I]MIBG uptake and striatal [^11^C]CFT uptake [30].

In the eight combined [^123^I]MIBG and DAT studies, the strength of the found correlation varied substantially. The observed variability in the presence and strength of within-subject correlation has multiple possible explanations. First, it may partly reflect methodological differences, including the use of either PET or SPECT and the region-of-interest definition for DAT imaging. Heterogeneity in disease stage and severity across studies may be less likely to explain the inconsistent results, as two studies found a correlation independently of disease stage, severity, and age [22,23]. Second, as the largest studies reported weak correlations, strong or absent correlations reported in smaller studies may reflect sampling variability rather than true biological differences. Importantly, two studies found a significant correlation in patients with an akinetic-rigid phenotype but not in those with a tremor-dominant phenotype, suggesting a stronger correlation between nigrostriatal dopaminergic degeneration and cardiac sympathetic denervation in akinetic-rigid PD.

The interpretation that the correlation of striatal dopaminergic degeneration and cardiac sympathetic denervation may be phenotype-specific is indirectly supported by unimodal imaging studies. Several [^123^I]MIBG studies have shown significant inverse correlations between myocardial uptake and the severity of hypokinesia, but not tremor [31,32], while [^123^I]FP-CIT studies similarly showed that striatal uptake inversely correlates with bradykinesia and rigidity rather than tremor [33,34]. Consequently, differences in the relative proportion of patients with tremor-dominant and akinetic-rigid PD included in each cohort may explain the observed variability in the presence and strength of reported correlations across studies. From a pathophysiological perspective, the brain-first subtype of PD, as proposed in the α-Synuclein Origin site and Connectome (SOC) model, is hypothesized to present more frequently with a tremor-dominant phenotype and relatively preserved cardiac sympathetic innervation at the onset of Parkinsonism [6,35]. A delayed involvement of the cardiac sympathetic nervous system relative to nigrostriatal dopaminergic degeneration in this subtype may therefore explain why correlations between these imaging biomarkers are weaker or absent in tremor-dominant PD.

Overall, the evidence supporting a correlation between nigrostriatal dopaminergic degeneration and cardiac sympathetic denervation remains limited, and both methodological heterogeneity and phenotypic variability in PD preclude a detailed understanding of this relationship.

### 4.3. Sensitivity–Specificity Trade-Off and Dependence on Patient Spectrum of Striatal Dopaminergic and Cardiac Sympathetic Imaging

Across the included studies, there was a wide range of sensitivities and specificities for both [^123^I]FP-CIT SPECT and [^123^I]MIBG scintigraphy when differentiating PD from the respective comparison groups, consisting predominantly of APS. In the subset of studies using pre-defined semi-quantitative thresholds, sensitivity was consistently higher for [^123^I]FP-CIT SPECT, whereas specificity was consistently higher for [^123^I]MIBG scintigraphy.

Why [^123^I]FP-CIT is more sensitive

The lower sensitivity of [^123^I]MIBG scintigraphy compared to [^123^I]FP-CIT SPECT is in line with reports finding a lower sensitivity for [^123^I]MIBG in early PD, since most studies in our review also included patients imaged during the diagnostic work-up. Ryu and colleagues found an increase in sensitivity for [^123^I]MIBG from 72% to 89% after a 2-year follow-up scan in 162 patients with PD [36], suggesting that sympathetic denervation may occur later in the disease course in some patients. In contrast, Marek and colleagues followed 91 patients with [^123^I]FP-CIT scans without evidence of dopaminergic deficit (SWEDD) for 22 months, and in 44% of patients, the diagnosis was eventually changed from PD to other disorders not associated with DAT deficit. This suggests that imperfect [^123^I]FP-CIT sensitivity in early PD is more likely to stem from clinical misclassification [9]. Recent studies also suggest that DAT PET may be associated with a lower rate of false-positive [37] and false-negative results [38] compared with [^123^I]FP-CIT SPECT.

Why [^123^I]MIBG can appear more specific

In the subset of five studies using pre-defined semi-quantitative thresholds, specificity was consistently higher for [^123^I]MIBG. However, the added value of [^123^I]MIBG scintigraphy may be overestimated since a large part of the available evidence relies on comparison groups consisting mostly or exclusively of APS. Given that [^123^I]FP-CIT is not suited for distinguishing PD from APS [8], while [^123^I]MIBG has a higher specificity for this differentiation [9,39], this patient spectrum inflates the relative specificity advantage of [^123^I]MIBG. This limits the applicability of these results to routine clinical settings where the differential diagnosis of suspected PD also includes non-neurodegenerative Parkinsonism. A 2023 systematic review of unimodal [^123^I]MIBG scintigraphy in PD found a pooled specificity of 80% [9], comparable to specificities of 80–100% reported for PD in unimodal [^123^I]FP-CIT studies [8], but comparison groups also varied widely in unimodal studies for both modalities, limiting comparability. The potential of PET imaging of cardiac sympathetic innervation with [^18^F]MFBG to improve diagnostic accuracy in PD and DLB compared with [^123^I]MIBG SPECT is currently under investigation; however, definitive results are not yet available.

Clinical implications of combined [^123^I]FP-CIT SPECT and [^123^I]MIBG scintigraphy

On average, studies reported a very high specificity for a combined positive result of both [^123^I]MIBG scintigraphy and [^123^I]FP-CIT SPECT, but at the cost of lower sensitivity compared to [^123^I]FP-CIT SPECT alone. Multivariable and model-based combinations show high diagnostic accuracies but are poorly generalizable and comparable due to retrospective case–control design with high risk of patient selection bias or post hoc optimization of thresholds.

Overall, the available evidence suggests higher sensitivity of [^123^I]FP-CIT in early PD while providing limited support for added specificity of [^123^I]MIBG in the differential diagnosis between PD and APS. This is more consistent with a role of [^123^I]MIBG in selected scenarios where APS are a consideration, as suggested by the unimodal [^123^I]MIBG literature [9], rather than with a complementary role of [^123^I]MIBG alongside [^123^I]FP-CIT in all patients with suspected PD. Local tracer availability and competing (imaging) techniques such as [^18^F]-FDG PET also play a role.

### 4.4. Limitations and Future Directions

Limitations of the within-subject correlation evidence base

The summary of evidence regarding the within-subject correlation of cardiac sympathetic and striatal dopaminergic imaging may be influenced by selective outcome reporting. Only two small studies reported an absence of correlation. However, several studies identified in this review acquired both cardiac sympathetic and striatal dopaminergic imaging primarily for diagnostic classification rather than to assess inter-system correlations. In such settings, correlation analyses may not have been systematically performed or reported when no significant association was observed. This could lead to underreporting of the absence of correlation in the published literature.

Limitations of the diagnostic accuracy evidence base

Most studies reporting diagnostic accuracies shared substantial sources of bias that limit the generalizability of the results. Four of the included studies determined imaging thresholds post hoc using ROC analysis on their own datasets. Such data-driven cutoffs are influenced by the specific composition of the comparison group, introduce a high risk of bias, and may limit the external validity of the results. Furthermore, all studies used the final clinical diagnosis as the reference standard. The timing of the clinical diagnosis relative to imaging was not reported in most studies, with only a subset reporting a clinical follow-up and only one explicitly reporting blinding to the molecular imaging results. A likely inconsistent temporal relationship between the clinical diagnosis and the index tests and a lack of blinding introduce a substantial risk of bias to the reported diagnostic accuracies.

Future directions

Future multimodal imaging studies should incorporate a detailed history of motor and non-motor symptoms to investigate differences in multimodal imaging profiles across clinical phenotypes and hypothesized pathophysiological subtypes. Regarding clinical use, detailed parameters for when dual imaging was used and pre-specified or externally validated semi-quantitative thresholds may reduce the risk of bias and improve the generalizability of diagnostic accuracy results. Analyses differentiating PD from APS and from non-neurodegenerative Parkinsonism should be reported separately to better characterize the added value of cardiac sympathetic imaging after striatal dopaminergic imaging in real clinical scenarios.

## 5. Conclusions

Most studies report a statistically significant within-subject correlation between nigrostriatal dopaminergic degeneration and cardiac sympathetic denervation, but the evidence base is heterogeneous, modest in size, and methodologically variable. Furthermore, there is limited evidence that the correlation depends on the clinical phenotype. In theory, this heterogeneity may imply a weak correlation and potentially complementary sensitivity across hypothesized PD subtypes. Multimodal imaging studies generally suggest a lower sensitivity and higher specificity of [^123^I]MIBG compared to [^123^I]FP-CIT in early PD. However, the apparent higher specificity of [^123^I]MIBG is inflated by comparison cohorts consisting predominantly of APS, and studies bear high risks of bias. The evidence base, therefore, does not sufficiently support routine complementary use of [^123^I]MIBG and [^123^I]FP-CIT in the evaluation of suspected PD.

## Figures and Tables

**Table 1:** Summary of studies reporting sensitivity and specificity for combined use of [^123^I]MIBG and [^123^I]FP-CIT in PD diagnosis. Combined approaches consistently improved specificity compared with DAT imaging alone, with variable effects on sensitivity.

Reference	*n* PD	Comparison Group	[^123^I]FP-CIT		[^123^I]MIBG		Combined Assessment		
			Sens	Spec	Sens	Spec	Sens	Spec	Combination Method
Treglia et al., 2014 [13]	40	16 MSA, 5 VP, 5 PSP, 2 CBS	95	21	80	75	78	79	Combined positivity
Yamada et al., 2016 [14]	63	4 PSP, 2 CBS, 1 MSA, 2 other	92	11	75	89	70	89	Combined positivity
Uyama, Naoto, 2017 [20]	15	5 MSA-P, 2 PSP, 12 with parkinsonism but no definite final diagnosis	87	53	93	47	87	74	Combined positivity
Yoshii et al., 2017 [12]	120	8 MSA, 10 PSP, 2 CBS	92	15	78	90	74	95	Combined positivity
Okada et al., 2018 [15]	37	11 initially suspected PD but no definite final diagnosis	78	73	65	100	81	91	SBR × H/M ratio
Matsusue et al., 2018 [18] (PD vs. APS)	36	4 PSP, 3 MSA, 1 CBS	81 *	63 *	92 °	63 °	92	63	Multiparametric scoring system: early H/M ratio, delayed H/M ratio, WR, average SBR, SBR asymmetry
Matsusue et al., 2018 [18] (PD vs. nPD, nAPS)		3 AD, 2 VP, 1 ET, 1 organophosphorous poisoning, 1 folic acid deficiency	69 **	75 **	83 °°	63 °°	83	88	
Stathaki et al., 2020 [16]	13	12 MSA, 4 DLB, 4 CBS, 2 ET, 2 dystonia, 4 other	100	38	82	79	82	92	Combined positivity
Iwabuchi et al., 2021 [19]	90	80 DIP/ET, 21 DLB, 16 PSP, 9 MSA	N/A	N/A	N/A	N/A	91	79	Classification and regression tree analysis: SBR, PCR, AI, early H/M ratio, late H/M ratio, WR

* based on SBR asymmetry, ** based on average SBR, ° based on delayed H/M ratio, °° based on washout rate Abbreviations: MSA-P multiple systems atrophy–Parkinsonian variant, VP vascular Parkinsonism PSP progressive supranuclear palsy, CBS corticobasal syndrome, AD Alzheimer’s dementia, ET essential tremor, DLB dementia with Lewy bodies, DIP drug-induced Parkinsonism, SBR specific binding ratio, H/M ratio heart-to-mediastinum ratio, WR washout rate, PCR putamen/caudate ratio, AI asymmetry index.

## Data Availability

No new data were created or analyzed in this study.

## Supplementary Materials

The following supporting information can be downloaded at: https://www.mdpi.com/article/10.3390/brainsci16050484/s1, Table S1: Subject characteristics, imaging modalities and uptake parameters of studies reporting within-subject correlations between nigrostriatal dopaminergic degeneration and cardiac sympathetic denervation in PD. Where reported, disease duration and Hoehn and Yahr stage are expressed as mean ± SD, median (IQR), or range, according to the original study. Binding ratio, specific binding ratio, and specific/non-specific binding ratio are reported using the terminology and definitions of the original studies. These metrics are not interchangeable. Abbreviations: H/M = Heart-to-mediastinum. Table S2: PD cohort characteristics, uptake parameters, thresholding methods and clinical diagnosis details of studies reporting sensitivity and specificity for a combined assessment with both [^123^I]MIBG and [^123^I]FP-CIT. Disease duration and Hoehn and Yahr stage are reported as mean ± SD. De novo = disease duration was not reported but study data was collected during first diagnostic workup. Binding ratio, specific binding ratio and uptake ratio are reported using the terminology and definitions of the original studies. These metrics are not interchangeable. Abbreviations: H/M Heart-to-mediastinum; MM(-PD) Mild-to-moderate; S(-PD) Severe; CART Classiciation And Regression Tree; ROC Receiver Operating Characteristic. * Use of the same software (DaTView, Nihon Medi-Physics, Tokyo, Japan) ° Use of Tossici-Bolt’s method for quantitative analysis

## Abbreviations

The following abbreviations are used in this manuscript:

PD	Parkinson’s disease
DAT	Dopamine transporter
MSA	Multiple system atrophy
PSP	Progressive supranuclear palsy
APS	Atypical Parkinsonian syndrome
NET	Norepinephrine transporter
SPECT	Single photon emission computed tomography
PET	Positron emission tomography
H/M ratio	Heart-to-mediastinum ratio
SBR	Specific binding ratio
DLB	Dementia with Lewy bodies
SWEDD	Scan without evidence of dopaminergic deficit
