# Supplementary material for: Combined Striatal Dopaminergic and Cardiac Sympathetic Imaging in Parkinson’s Disease
Source: Brain Sci. 2026 Apr 30;16(5):484. doi: 10.3390/brainsci16050484 (PMC13204103; doi:10.3390/brainsci16050484)
Supplement: Supplementary file 1 [file brainsci-16-00484-s001.zip › brainsci-4096505-supplementary.pdf]

Reference	PD cohort characteristics			Imaging modality		Uptake parameter	
	n	PD disease duration (years)	H&Y stage	Striatal dopaminergic	Cardiac sympathetic	Striatal dopaminergic	Cardiac sympathetic
Spiegel et al., 2005	19	2.4±2.2	1	[^{123}I]FP-CIT SPECT	[^{123}I]MIBG scintigraphy	Caudate and putamen binding ratio (occipital lobe reference)	H/M ratio
Raffel et al., 2006	9	3–9	1.5–2.5	[^{11}C]DTBZ PET	[^{11}C]HED PET	Caudate and anterior putamen distribution volume ratio (occipital cortex reference)	Left ventricle tracer retention
Spiegel et al., 2007	95	Unclear	I: 63, II: 12 III+IV: 20	[^{123}I]FP-CIT SPECT	[^{123}I]MIBG scintigraphy	Caudate and putamen binding ratio (occipital cortex reference)	H/M ratio
Ishibashi et al., 2010	16	3.5±3.2	2.4±0.9	[^{11}C]CFT PET	[^{123}I]MIBG scintigraphy	Caudate, anterior- and posterior putamen uptake ratio index (cerebellum reference)	H/M ratio
Chiaravalloti et al., 2013	37	2.2±1.9	1.7±0.7	[^{123}I]FP-CIT SPECT	[^{123}I]MIBG scintigraphy	Caudate and putamen specific binding ratio (occipital cortex reference)	H/M ratio
Asahi et al., 2016	15	Unclear	2.9±0.9	[^{123}I]FP-CIT SPECT	[^{123}I]MIBG scintigraphy	Striatum specific binding ratio (whole brain reference)	H/M ratio
Yoshii et al., 2017	120	6.7±5.5	2.6±0.8 ("on" state)	[^{123}I]FP-CIT SPECT	[^{123}I]MIBG scintigraphy	Striatum specific binding ratio (whole brain reference)	H/M ratio
Oh et al., 2019	96	Normal H/M ratio group: 1.2±0.8; Decreased H/M ratio group: 1.2±1.2	Normal H/M ratio group: 1.8±0.6; Decreased H/M ratio group: 1.7±0.6	[^{18}F]FP-CIT PET	[^{123}I]MIBG scintigraphy	Caudate, putamen and pallidum SUVR (cerebellar reference region)	H/M ratio
Jang et al., 2021	31	2.3±2.0	1–2	[^{18}F]FP-CIT PET	[^{123}I]MIBG scintigraphy	Caudate and lenticular specific/non-specific binding ratio (occipital cortex reference)	H/M ratio
Ozawa et al., 2024	62	1 (1–2)	2 (2–2)	[^{123}I]FP-CIT SPECT	[^{123}I]MIBG scintigraphy	Lenticular specific binding ratio (reference region not specified)	H/M ratio

Supplementary table 1. Subject characteristics, imaging modalities and uptake parameters of studies reporting within-subject correlations between nigrostriatal dopaminergic degeneration and cardiac sympathetic denervation in PD. Where reported, disease duration and Hoehn and Yahr stage are expressed as mean±SD, median (IQR), or range, according to the original study. Binding ratio, specific binding ratio, and specific/non-specific binding ratio are reported using the terminology and definitions of the original studies. These metrics are not interchangeable. Abbreviations: H/M = Heart-to-mediastinum

Reference	PD cohort characteristics			Striatal [^{123}I]FP-CIT SPECT			Cardiac [123I]MIBG scintigraphy			Clinical diagnosis			Interfering drugs avoided?
	n	Disease duration (years)	PD clinical severity (H&Y stage)	Uptake parameter	Parameter threshold	Threshold determination method	Uptake parameter	Parameter threshold	Threshold determination method	Timing of diagnosis	Clinical follow-up?	Medication response documented?	
Treglia et al., 2014	40	De novo	Unclear	Striatal binding ratio (posterior striatum reference)	<2.6=PD	>2 SD below internal healthy control group	H/M ratio	<1.55=PD	>2 SD below internal healthy control group	Unclear	No	No	Unclear
Yamada et al., 2016	63	6.3±5.8	3.1±0.9	Striatal specific binding ratio (whole-brain reference)°	≤4.5=PD	Taken from literature	H/M ratio	<2.2=PD	Taken from literature	Unclear	No	No	Yes
Uyama et al., 2017	15	De novo	Unclear	Striatal specific binding ratio (whole-brain reference)*°	<3.24=PD	ROC analysis on study dataset	H/M ratio	<2.745=PD	ROC analysis on study dataset	Unclear	No	Yes	No
Yoshii et al., 2017	120	6.7±5.5	2.6±0.8 ('on' state)	Striatal specific binding ratio (whole-brain reference)*°	<4.5=PD	ROC analysis on study PD dataset versus internal healthy control dataset	H/M ratio	<2.2=PD	Taken from literature	Unclear	Yes (retrospectively recruited after FU)	Yes	Yes
Okada et al., 2018	37	De novo	Unclear	Striatal specific binding ratio (whole-brain reference)* °	<3.8=PD	Taken from literature	H/M ratio	<2.2=PD	Taken from literature	Unclear	No	No	Yes
Matsusue et al., 2018	36	MM-PD: 1.2±1.3; S-PD: 2.4±2.5	2.6±1.2	Striatal specific binding ratio (whole-brain reference)*°	<4.29=PD	ROC analysis on study dataset	H/M ratio and washout rate	H/M ratio: <1.9=PD; Washout rate: >19%=PD	ROC analysis on study dataset	Unclear	Yes (retrospectively recruited after FU)	Yes	Yes
Stathaki et al., 2020	13	De novo	Unclear	Caudate and putamen uptake ratio (occipital cortex reference)	Unclear	Unclear	H/M ratio	<1.43=PD	Taken from literature	2-5 years after first visit to study clinic	Yes, 2-5 years	Yes	Yes
Iwabuchi et al., 2021	90	De novo	Unclear	Specific binding ratio (occipital lobe reference), putamen/caudate ratio, asymmetry index*	N/A (multiparametric decision tree)	CART analysis on study dataset	H/M ratio and washout rate	N/A (multiparametric decision tree)	CART analysis on study dataset	Unclear	Yes (retrospectively recruited after FU)	Yes	Yes

Supplementary table 2: PD cohort characteristics, uptake parameters, thresholding methods and clinical diagnosis details of studies reporting sensitivity and specificity for a combined assessment with both [^{123}I]MIBG and [^{123}I]FP-CIT. Disease duration and Hoehn and Yahr stage are reported as mean ± SD. De novo = disease duration was not

reported but study data was collected during first diagnostic workup. Binding ratio, specific binding ratio and uptake ratio are reported using the terminology and definitions of the original studies. These metrics are not interchangeable. Abbreviations: *H/M* Heart-to-mediastinum; *MM(-PD)* Mild-to-moderate; *S(-PD)* Severe; *CART* Classiciation And Regression Tree; *ROC* Receiver Operating Characteristic. ***** Use of the same software (DaTView, Nihon Medi-Physics, Tokyo, Japan) ° Use of Tossici-Bolt's method for quantitative analysis.
